# Artificial intelligence in esophageal cancer: a narrative review from a thoracic surgical perspective

**DOI:** 10.3389/fonc.2026.1917622

**Published:** 2026-10-20

**Authors:** Dongrong Peng, Wenyi Liu, Baihua Zhang

**Affiliations:** 1 Department of Thoracic Surgery, National Cancer Center/National Clinical Research Center for Cancer/Cancer Hospital & Shenzhen Hospital, Chinese Academy of Medical Sciences and Peking Union Medical College, Shenzhen, China; 2 Department of Thoracic Surgery, Fujian Medical University Union Hospital, Fuzhou, China

**Keywords:** artificial intelligence, clinical decision support, deep learning, esophageal cancer, esophagectomy, radiomics, thoracic surgery

## Abstract

**Background:**

Artificial intelligence (AI) has potential applications across the esophageal cancer (EC) care pathway, but reported retrospective performance often has not translated into clinically validated decision support. We review the evidence through the lens of thoracic surgical decisions, where the consequences of prediction errors are asymmetric.

**Scope:**

This narrative review examines AI studies relevant to thoracic surgical decisions, with an emphasis on esophageal squamous cell carcinoma (ESCC). Selected reports were mapped to clinical tasks, analysis units, and validation settings. Supplementary PubMed and Web of Science searches informed the review; studies were selected purposively to illustrate clinical tasks and validation problems, not to represent the entire field.

**Key observations:**

A randomized endoscopy trial supports improved lesion detection in its study setting. Selected retrospective studies report external testing for nodal risk or pathological response, whereas surgical video and complication models mainly provide internal evaluation. Image-level results, patient-level predictions, and clinical outcomes are distinct. Calibration and prospective pathway evaluation remain necessary before treatment allocation can be based on model output.

**Conclusions:**

AI in EC should presently be considered an investigational decision-support layer rather than a replacement for endoscopic, imaging-based, or multidisciplinary assessment. Translation requires patient-level data partitioning, transparent reporting of calibration and clinical utility, independent external validation, and prospective studies using prespecified decision thresholds.

## 1 Introduction

According to GLOBOCAN 2020 estimates, esophageal cancer (EC) ranked seventh in global cancer incidence and sixth in cancer-related mortality, accounting for approximately 604,000 new cases and 544,000 deaths in 2020 (1). The two principal histological subtypes have distinct epidemiology: esophageal squamous cell carcinoma (ESCC) predominates globally and is particularly prevalent in East Asia, whereas esophageal adenocarcinoma (EAC) is the dominant subtype in several Western populations and is associated with Barrett’s esophagus (2–4). Overall five-year survival remains poor and varies materially by stage, geography, histology, and treatment access; historical population-level estimates are commonly in the 15-25% range (5).

Several clinical challenges motivate research on artificial intelligence (AI) in EC. Early-stage disease can be asymptomatic, and preoperative assessment remains imperfect for invasion depth and nodal involvement (3, 4). Treatment selection depends on stage, histology, patient fitness, and the planned multimodal regimen; response prediction must therefore be evaluated within a defined treatment pathway rather than assumed to transfer across all resectable EC. Esophagectomy carries substantial morbidity, with overall complication rates approaching 65% in a nationwide cohort (6, 7). TNM staging remains essential but does not fully account for outcome variation among patients within the same stage (8).

AI, particularly the deep-learning (DL) approaches that now dominate medical image analysis, offers methods for extracting quantitative patterns from multidimensional data (9). AI applications in EC have proliferated across the care pathway—computer-aided detection in endoscopy, radiomic prediction of treatment response, intraoperative video analytics, postoperative complication prediction, and prognostic modeling (10–12). A substantial gap nonetheless persists between reported AI performance metrics and demonstrated clinical utility in routine practice.

This narrative review uses selected AI studies to examine questions relevant to thoracic surgical care in EC. It follows the clinical pathway from detection to follow-up (**Figure 1**), with particular attention to four decisions: additional treatment after endoscopic resection, surgery versus active surveillance after clinical complete response, lymphadenectomy extent, and selection for salvage surgery. We distinguish model discrimination from evidence that an AI-supported pathway improves decisions or outcomes. The decision-centered framework is an author-proposed conceptual tool for organizing research priorities, not a validated evidence-grading system or clinical treatment rule.

**Figure 1:**
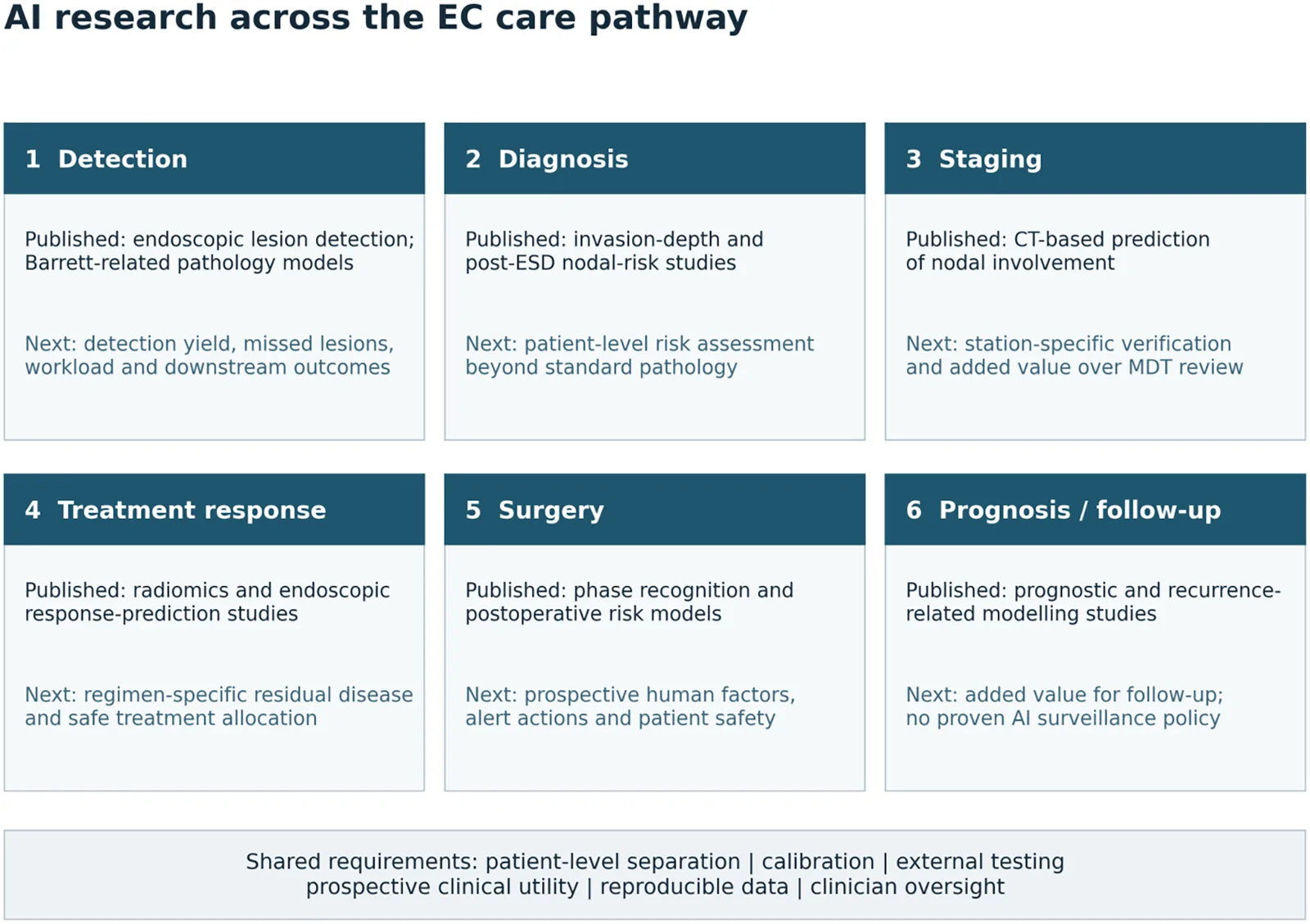
AI research across the esophageal cancer care pathway. Each panel separates published application examples from questions that require prospective evaluation. The presence of a published model does not imply clinical readiness. Detection, response assessment, and postoperative surveillance are distinct tasks; baseline metastatic-status classification is not evidence of recurrence surveillance. Shared methodological requirements apply throughout the pathway.

## 2 Scope, search strategy and study selection

This narrative review examines AI applications relevant to thoracic surgical decision-making in esophageal cancer, with an emphasis on ESCC reflecting the authors’ East-Asian clinical practice context. EAC and Barrett-related neoplasia are included where clinically relevant, particularly in early detection and response assessment. Evidence is synthesized qualitatively by clinical task rather than pooled across heterogeneous endpoints and validation settings.

To update the review, supplementary searches of PubMed and Web of Science were conducted on 6 September 2026. Broad AI searches covered publication dates from 1 January 2015 to 6 September 2026, with additional AI and clinical-context searches covering 1 April to 6 September 2026. Search terms addressed esophageal cancer and related lesions, AI methods, neoadjuvant immunotherapy, circulating tumor DNA (ctDNA) and surveillance. English-language reports were considered; full strategies, retrieval counts and screening details are provided in the **Supplementary Methods** and **Supplementary Table S1**.

Reports were considered across detection, staging, treatment response, surgical management, prognosis, surveillance and related radiotherapy workflows. Mixed-cancer studies required separately interpretable results for the population of interest. Treatment, ctDNA and organ-preservation studies provided clinical context; reviews and methodological guidance supported interpretation. Protocols, conference abstracts without a full journal report, corrections and non-clinical experiments were not treated as independent completed clinical AI validation studies.

**Table 1** comprises 14 illustrative studies selected for clinical relevance, task coverage, source accessibility and informative validation features. This author-directed selection supports comparison of clinical questions and methodological limitations; it is not a representative sample of the field, an exhaustive evidence map or a ranking by performance. Source availability and the questions emphasized by the authors may influence which studies are included, so the table cannot establish how common a finding or limitation is across EC AI research. Reports were examined for design, analysis unit, cohort allocation and model-specific results. **Supplementary Table S2** records selection reasons, source-check scope and author review. English-language selection, the ESCC emphasis and incomplete source access introduce additional selection limitations. Generative AI assistance is disclosed in the **Supplementary Methods** and Acknowledgments.

**Table 1:** Fourteen representative AI studies mapped to clinical tasks, analysis units, validation settings, and limitations.

Study and task	Target and model	Design, analysis units and data partitioning	Reported evaluation result	Validation setting and interpretation
Li 2024 (14) Screening	Detection of high-risk esophageal lesions, including low-/high-grade intraepithelial neoplasia and cancer ENDOANGEL-ELD; real-time CNN system	Prospective single-center trial; patient-level endpoint; electronic 1:1 randomization, blocks of four 3,117 patients aged ≥50 years: assisted 1,556; control 1,561. Patients, data collectors and analysts blinded; endoscopists unblinded. System development used 191,744 esophagogastroscopic images from three centers; detailed patient counts and development partitions were not verified.	Intention-to-treat HrEL detection: 28/1,556 (1.8%) versus 14/1,561 (0.9%); P = 0.029.	Full report checked: Table 2 of Li et al. (14) and Methods. Clinical trial at one center despite multicenter model-development images. Non-biopsied negative examinations limit diagnostic-accuracy assessment. Supplementary development details unavailable; no survival or surgical-outcome benefit established.
Yuan 2022 (15) Endoscopic diagnosis	Superficial ESCC detection YOLOv3-based deep convolutional neural network	Retrospective multicenter; image and video Development 45,770 images/2,244 patients; internal 6,075/277; external 2,088/100 patients from 4 hospitals; 142 videos/19 patients	External image test: accuracy 91.2%, sensitivity 97.2%, specificity 82.3%, AUC 0.946	Four-hospital external image test. Internal evaluation used a later period. Patient counts and image-level performance are distinct; video results do not establish patient-level clinical benefit.
Ren 2025 (22) Histopathology	LNM prediction in T1 ESCC Hierarchical GNN	Retrospective; WSI, with case-level results separately reported 160 patients: development/validation 112 (442 WSIs), internal test 48 (217 WSIs); external ESD 35 (161 WSIs), four LNM-positive patients	Internal AUC 0.949; external slide accuracy 90.1%, NPV 96.9%; external case sensitivity 4/4, specificity 26/31, NPV 26/26	Separate ESD cohort; 35 patients. Small-event external test. Nodal reference: surgery or follow-up. Cutoff selected on internal test set. Case and slide estimates differ.
Bouzid 2024 (20) Histopathology	Barrett’s esophagus detection from H&E WSI Weakly supervised DL	Retrospective computational pathology; patient cohorts with WSI inputs DELTA: 1,141 patients (912 development, 229 test); external BEST2: 725 patients.	DELTA test AUC 0.914; BEST2 external AUC 0.873 (95% CI 0.843–0.900).	Separate BEST2 cohort. Barrett detection, not invasive cancer diagnosis. Workload reduction was simulated rather than measured in prospective implementation.
Shen 2018 (26) Staging	Preoperative LNM prediction CT radiomics signature; a combined nomogram was also developed.	Single-center retrospective temporal split; patient 197 patients: training 140, validation 57	Radiomics signature validation AUC 0.771 (95% CI 0.632-0.910); not interchangeable with combined nomogram performance	Temporal internal validation. DCA was reported, but its threshold range is not a clinically validated decision cut-off.
Chen 2022 (27) Staging	LNM prediction in ESCC Handcrafted + ResNet50 deep features + clinical RF	Single-center retrospective; patient-level split, node CT features 308 patients: training 216, test 92	RF Model III test AUC 0.80 (95% CI 0.71–0.89); sensitivity and specificity both 0.76.	Internal testing; model selection across configurations. Best result selected across algorithm/feature combinations, not a separately validated locked model. Node-derived features do not establish individual-node pathological matching.
Li 2026 (28) Nodal staging	Pathologic N2+ ESCC prediction from preoperative CT Self-supervised 3D deep learning	Retrospective, two-center; 1,060 patients: development 612 (428/92/92 training/validation/internal test), temporal test 238, external test 210. Patient-disjoint; overlapping patients excluded from 3,200 pretraining scans.	External AUC 0.860 (95% CI 0.810–0.906); sensitivity 0.581, specificity 0.845; Brier 0.148, ECE 0.053.	Geographic external and temporal testing. Validation-only tuning/calibration reported. Predicts nodal burden, not station-specific involvement; does not validate reduced lymphadenectomy.
Kasai 2024 (42) Treatment response	CRT complete response maintained >1 year CT radiomics random forest (12 features)	Retrospective; patient-level cohorts from different departments of the same hospital 44 patients: training 27 (6 responders), validation 17 (6 responders).	Validation AUC 0.92 (95% CI 0.71–0.99).	Same-hospital departmental validation. Sustained complete response of the primary tumor, not surgical pCR. Few response events; high AUC alone proves neither overfitting nor clinical utility.
Xie 2023 (43) Treatment response	Radiographic RT response (>50% reduction) Attention ResNet50 radiomics	Multicenter retrospective; 248 patients: training 154, internal validation 45, external validation 49. Multiple images per patient; patient-level aggregation of image predictions is unclear.	Reported AUC: internal 0.802; external 0.732. Patient-level aggregation remains unconfirmed.	External cohort testing. Radiographic response, not pCR. Image augmentation does not increase the number of independent patients.
Hu 2020 (35) Treatment response	pCR to nCRT Intratumoral + peritumoral CT radiomics (RBF-SVM)	Two-center retrospective CT-radiomics; patient 231 ESCC after nCRT and surgery: development 161, external test 70	External AUC 0.852 (95% CI 0.753–0.951).	External cohort testing. pCR assessed in resection specimens. External testing does not validate surgery omission. Separation of tuning and harmonization from test data is unclear.
van Jaarsveld 2026 (48) Intraoperative surgery	RAMIE anatomy and instrument segmentation; phase recognition Deep learning	Retrospective, single-center; anatomy: 1,504 frames from 53 videos (train/validation/test 1,025/137/342 frames), with video-disjoint partitions. Phases: 38 videos, patient-level split 21/6/11.	Segmentation Dice 0.72 across anatomical and instrument classes; phase-recognition accuracy 82.8%.	Internal testing only. Anatomy and phase datasets must not be summed as unique patients. Task-specific results; no demonstrated reduction in surgical complications.
van de Beld 2024 (55) Postoperative complications	Prediction of anastomotic leak and pneumonia Temporal unimodal and multimodal ML compared.	Single-center retrospective; serial postoperative inputs; patient 417 esophagectomy patients (2011-2021); repeated fourfold CV	Best reported one-day-ahead AUC: leak 0.87; pneumonia 0.74. The leak result belongs to the temporal laboratory model, not multimodal fusion.	Internal repeated cross-validation. Lead time is relative to complication diagnosis, not postoperative day 1. Imaging also contributes to pneumonia labels; input timing and outcome definition matter.
Jung 2023 (56) Postoperative complications	Major, medical and surgical complications Neural network	Single-center retrospective; 864 patients Threefold CV, approximately 66% training and 33% testing in each fold.	AUC 0.672, 0.695 and 0.653, respectively	Internal threefold cross-validation. The workflow figure places imputation and collinearity processing before fold assignment, raising concern about potential preprocessing leakage; implementation-level leakage is not established. No separate institution identified.
Wan 2024 (29) Metastasis prediction	Hepatic metastasis at diagnosis Gradient boosting machine	Retrospective SEER registry model; patient 17,800 patients (2010-2020): training 12,460, internal test 5,340; 10-fold CV in training	Internal-test AUC 0.885, accuracy 0.868, sensitivity 0.667, specificity 0.888	Random internal split. Inputs include concurrent bone/lung metastasis and recorded treatments; it is not a validated prospective surveillance-allocation tool.

Selection is purposive; **Supplementary Table S2** records study-specific reasons and source-check status. The table is not exhaustive or ranked by AUC. Patient, image, slide and video results are not interchangeable. Patient-disjoint cohorts do not themselves establish patient-level performance aggregation. A separate cohort is not necessarily an independent hospital or prospective clinical pathway. Source-check scope is documented in **Supplementary Table S2**; unclear methods are not assumed to be absent. The Li randomized detection entry was checked against the full report, particularly Table 2 of Li et al. (14) and the trial methods; **Supplementary Materials** were not available. Its primary endpoint includes low- and high-grade intraepithelial neoplasia as well as cancer. AUC, area under the ROC curve; CNN, convolutional neural network; CRT, chemoradiotherapy; CT, computed tomography; CV, cross-validation; DCA, decision-curve analysis; DL, deep learning; ECE, expected calibration error; ESD, endoscopic submucosal dissection; ESCC, esophageal squamous cell carcinoma; GNN, graph neural network; H&E, hematoxylin and eosin; HrEL, high-risk esophageal lesion; LNM, lymph node metastasis; ML, machine learning; nCRT, neoadjuvant chemoradiotherapy; NPV, negative predictive value; pCR, pathological complete response; RAMIE, robot-assisted minimally invasive esophagectomy; RBF-SVM, radial-basis-function support vector machine; RF, random forest; RT, radiotherapy; SEER, Surveillance, Epidemiology, and End Results; WSI, whole-slide image.

## 3 AI in endoscopic and histopathological diagnosis

### 3.1 Detection and assessment of early lesions

AI-assisted endoscopic detection has been extensively reviewed (10, 13). In a single-center block- randomized trial involving 3,117 patients aged at least 50 years, Li et al. reported detection of high-risk esophageal lesions (HrELs) in 28 of 1,556 patients receiving convolutional neural network (CNN)-assisted endoscopy and 14 of 1,561 controls (1.8% versus 0.9%; P = 0.029) (14). The full report confirms this patient-level intention-to-treat endpoint [Table 2 of Li et al. (14)]. Randomization used an electronic system with blocks of four; patients, data collectors and analysts were blinded, but endoscopists knew allocation. Some negative examinations were not biopsied, limiting interpretation of the separately reported diagnostic accuracy. The absolute difference was approximately 0.9 percentage points, or about nine additional patients with HrELs detected per 1000 examinations in this trial. HrELs included low- and high-grade intraepithelial neoplasia as well as cancer; this is not nine additional cancers detected. Yuan et al. used a YOLOv3-based network, with external image evaluation distinct from patient-level clinical effectiveness (15). Other studies address early ESCC and Barrett-related neoplasia using different modalities (16–19). **Table 1** separates randomized detection evidence from image-based model evaluation; detection accuracy should not be interpreted as invasion-depth or nodal-risk accuracy.

Bouzid et al. evaluated Barrett detection from Cytosponge hematoxylin and eosin (H&E) slides, using 912 DELTA patients for development and 229 for testing, with 725 patients in the separate BEST2 cohort (20). The DELTA test area under the receiver operating characteristic curve (AUC) was 0.914 and the external AUC was 0.873 (95% CI 0.843–0.900). This non-endoscopic sampling application addresses screening triage rather than invasive cancer diagnosis; its workload estimates were simulated rather than measured during prospective implementation.

Detection and depth assessment answer different treatment questions. Endoscopic treatment selection requires a specified pathological depth boundary and integration of endoscopic findings with pathological risk features, staging and patient fitness (3, 4, 13). An AI detector cannot be assumed to classify invasion depth. Depth-assessment research should compare its added value with expert assessment at the boundary that would change management.

### 3.2 Lymph node risk prediction

In T1 ESCC, the possibility of lymph-node metastasis makes the choice between endoscopic resection with surveillance and esophagectomy clinically nuanced. Risk is heterogeneous and depends on pathological features such as invasion depth, lymphovascular invasion, differentiation, and tumor budding (21). Prediction tools should therefore be evaluated against these established features rather than used to replace them.

Ren et al. developed a whole-slide image (WSI)-based hierarchical graph neural network using 160 surgical cases, with patient-level partitioning into 112 development/validation cases and 48 internal-test cases (22). The external cohort after endoscopic submucosal dissection (ESD) comprised 35 patients and 161 slides, including four patients with lymph-node metastasis (LNM). External slide-level accuracy was 90.1% and negative predictive value (NPV) was 96.9%. With case-level aggregation, sensitivity was 4/4 and NPV was 26/26. These small denominators do not establish a reliable treatment-omission rule. The external nodal reference combined surgical pathology and follow-up, and the report states that the operating cutoff was selected using the internal test set; both features require consideration when interpreting validation.

Predictive values depend on prevalence and require a clearly stated analysis unit and uncertainty interval. For a positive label of LNM, 1 minus NPV is the false omission rate among model-negative observations; the false-negative rate is 1 minus sensitivity among LNM-positive observations. Neither a slide-level NPV nor the absence of missed positive cases in a four-event sample establishes patient-level safety. Multiple slides from one patient are correlated and must remain within a single data partition. Evaluation before treatment allocation should use a locked model and aggregation rule, an independent patient cohort, and a clinically appropriate nodal reference standard.

## 4 AI in staging and treatment planning

### 4.1 Radiomics for staging and lymph node assessment

Accurate preoperative staging in EC is limited by the modest sensitivity and specificity of conventional computed tomography (CT) and endoscopic ultrasonography (EUS) for micrometastatic nodal disease and for fine distinctions in T staging. Radiomics—the high-throughput extraction of quantitative features from medical images—has been developed to address these limitations by capturing texture, intensity, and shape information not assessable by visual inspection (23, 24). General medical-imaging AI literature further emphasizes that reproducible preprocessing, data partitioning, and validation are prerequisites for translating such image-derived models into clinical workflows (25).

Shen et al. developed a CT-radiomics signature and a combined nomogram for preoperative LNM prediction (26). The validation AUC of 0.771 (95% CI 0.632–0.910) refers to the signature, not the combined nomogram; the 140/57-patient split was temporal but within one center. Chen et al. reported an AUC of 0.80 (95% CI 0.71–0.89) for RF Model III in 92 test patients after development in 216 patients (27). This was the best result among compared model configurations, not a separate prospective evaluation of a locked model. Neither study establishes superiority over routine multidisciplinary staging.

Li et al. evaluated self-supervised CT prediction of pathologic N2+ disease in 1,060 patients, including temporal and geographic external test cohorts (28). External AUC was 0.860, with sensitivity 0.581 at the reported operating point. The endpoint concerns overall nodal burden, not individual stations. Calibration measures are reported in **Table 1**; these retrospective results do not establish that a negative result permits reduced lymphadenectomy.

Wan and Zeng developed a retrospective Surveillance, Epidemiology, and End Results (SEER)-based machine-learning model for hepatic metastasis at diagnosis in 17,800 patients (29). The gradient-boosting model achieved an AUC of 0.885 in a random internal test set (n=5,340). This result is not independent external validation, and the inputs included concurrent bone/lung metastatic status and recorded treatments; it should therefore be interpreted as registry-based risk classification rather than prediction of future recurrence. The timing of recorded treatment variables also limits its interpretation as a pretreatment decision aid.

AI-versus-radiologist comparisons require attention to the information available to each comparator. If a model incorporates clinical variables while readers assess images alone, the comparison does not isolate incremental benefit over routine multidisciplinary assessment. Many staging models predict patient-level nodal status rather than station-specific involvement. For surgical planning, a station-specific output may be more relevant, but it must be linked to pathological verification and the consequences of altering the dissection field.

### 4.2 Treatment response prediction and assessment

Response prediction must be anchored to histology and regimen. CROSS established the benefit of neoadjuvant chemoradiotherapy (nCRT) followed by surgery compared with surgery alone in its study population (30, 31). In resectable esophageal adenocarcinoma, ESOPEC subsequently demonstrated superior overall survival with perioperative FLOT compared with CROSS-based preoperative chemoradiotherapy (32). Neither result establishes one uniform pathway for all EC. Models developed after definitive chemoradiotherapy (CRT), nCRT, neoadjuvant chemotherapy, or chemoimmunotherapy answer different questions and require separate validation before transfer between settings.

#### 4.2.1 Pretreatment prediction of neoadjuvant response

Radiomics-based response prediction has been explored using CT and positron emission tomography/computed tomography (PET/CT) (11, 33, 34). Reported performance varies with treatment, response definition, acquisition, and validation design. Differences between pooled estimates from separate modality-specific study sets cannot by themselves establish that one modality is superior or explain the difference biologically. A treatment-allocation model should be assessed against standard response evaluation in the same patients and distinguish radiographic response from pathological response.

Hu et al. evaluated intratumoral and peritumoral CT radiomics in 231 ESCC patients treated with nCRT and surgery, using 161 patients for development and 70 for external testing (35). The external AUC was 0.852 (95% CI 0.753–0.951), with pathological complete response (pCR) assessed in resection specimens. This supports retrospective discrimination, not a validated rule for omitting surgery. Wang et al. explored radiomics in a neoadjuvant immunotherapy setting (36); transfer across regimens requires separate assessment.

#### 4.2.2 Post-treatment assessment and residual disease

Matsuda et al. reported a retrospective proof-of-concept study of endoscopic response after neoadjuvant chemotherapy in 193 ESCC patients (37). Evaluation used a 20-image test set with median performance across 10 models. This small image-based evaluation provides limited precision, and patient-level independence remains unclear. Its post-treatment endoscopic responder endpoint differs from pretreatment prediction of resection-specimen pCR; the two approaches cannot be substituted for each other in organ-preservation decisions.

Neoadjuvant immune checkpoint inhibition makes regimen-specific response assessment particularly important. In the phase 3 ESCORT-NEO/NCCES01 trial, pCR rates were 28.0% with camrelizumab plus nab-paclitaxel/cisplatin, 15.4% with camrelizumab plus paclitaxel/cisplatin, and 4.7% with paclitaxel/cisplatin alone (38). These pathological-response results do not establish that surgery can be omitted. Before resection, a clinical complete response (cCR) remains an assessment based on available tests rather than confirmation of pCR. The preSANO study evaluated a combined approach after nCRT using endoscopic sampling, EUS-guided sampling of suspicious nodes, and PET/CT to assess interval metastatic disease (39). ctDNA may provide complementary residual-disease or recurrence-risk information (40, 41), but a negative assay is not an established stand-alone rule-out test. Research on multimodal AI should test added value over standard response evaluation, report primary-tumor and nodal residual disease separately, and validate timing and performance within the actual treatment regimen. Evidence for active surveillance after nCRT cannot be directly extrapolated to chemoimmunotherapy. Atypical response patterns, including pseudoprogression, can complicate conventional imaging assessment during immunotherapy (36). Pretreatment response prediction and post-treatment residual-disease assessment should therefore be treated as separate tasks: a baseline CT model does not demonstrate an ability to distinguish treatment-related changes from viable tumor after therapy. Models developed in chemotherapy or chemoradiotherapy cohorts should undergo regimen-specific external validation and calibration before application to chemoimmunotherapy cohorts.

#### 4.2.3 Radiotherapy and definitive chemoradiotherapy settings

High discrimination requires interpretation in the context of the endpoint and validation design. Kasai et al. studied 44 patients split between departments of the same hospital (27 training, 17 validation); the target was primary-tumor complete response maintained for more than one year, not surgical pCR (42). Xie et al. evaluated radiographic response with training, internal and external cohorts of 154, 45 and 49 patients (43). Their reported external AUC of 0.732 should not be described as patient-level performance without confirming image-to-patient aggregation. General checks for overfitting and data leakage are discussed in Section 7.7.

Yin et al. studied a radiomics–clinical model in a definitive CRT setting (44). These findings concern response in a non-neoadjuvant pathway, rather than pCR verified after planned resection. Radiotherapy-related prognostic and molecular modeling is discussed separately in Section 6.

## 5 AI in surgical management of esophageal cancer

The thoracic surgical perspective here concerns the perioperative decisions to which AI might contribute, rather than established intraoperative deployment. The selected studies include anatomical and phase recognition, a small video-observer evaluation of human performance, and perioperative risk prediction. They do not establish clinical benefit from AI-guided selection of esophagectomy, organ preservation, dissection extent or salvage surgery. The following sections distinguish task performance, assistance to clinicians and changes in patient management.

### 5.1 Intraoperative AI in robot-assisted esophagectomy

Esophagectomy remains the cornerstone of curative treatment for resectable EC. Over the past decade, surgical practice has shifted toward minimally invasive and robot-assisted approaches. RAMIE provides three-dimensional visualization, articulating instrumentation, and reduced pulmonary morbidity relative to open surgery, but it is associated with a substantial learning curve and demands consistent technical performance from the operative team (45, 46). The combination of high procedural complexity and high clinical stakes makes RAMIE a priority application for intraoperative AI support.

Takeuchi et al. reported phase-recognition accuracy of 84% using 31 RAMIE cases and fourfold cross-validation (47). Patient-disjoint frame allocation is unclear, limiting interpretation of this proof-of-concept result. The newer van Jaarsveld study reported video-disjoint anatomy partitions and a patient-level phase split, with phase-recognition accuracy of 82.8% (48). These accuracies cannot be ranked across studies with different phase definitions and evaluation designs. Neither establishes a universal threshold for clinical deployment or a reduction in operative complications.

Recognition of critical structures such as the recurrent laryngeal nerve, thoracic duct, and vagal branches is a clinically relevant research target during esophagectomy (49). A useful system would need to identify structures reliably despite blood or smoke obscuring the field, traction-induced tissue deformation, anatomical variation, and differences in operative technique. Existing videos can support annotation, but expert agreement, visibility, temporal consistency, and cross-center validation must be established. Evaluation should report performance separately in these difficult conditions, including variant nerve or thoracic-duct anatomy, rather than relying only on overall accuracy.

Furube et al. provide an initial link between anatomical recognition and human performance (50). Eight trainee surgeons viewed five original and AI-enhanced RAMIE videos more than four weeks apart. For right recurrent laryngeal nerve (RLN) recognition, the reported times to identifying a candidate and confirming the nerve were 134 versus 178 seconds and 233 versus 325 seconds with versus without AI, respectively (both P<0.001). This small video-observer study supports investigation of recognition assistance, not a reduction in operative nerve injury. Repeated viewing and the small case set limit generalization to operative performance.

Fusion of preoperative three-dimensional reconstruction with intraoperative AI could eventually support augmented-reality navigation in thoracic surgery. A plausible research workflow is segmentation of patient-specific mediastinal anatomy and tumor on preoperative imaging, creation of a three-dimensional model, and registration to the operative field. Deformable registration remains a major technical challenge because positioning, lung deflation, and tissue mobilization alter the surgical field. At present, this is a research concept in EC surgery rather than an established clinical application; future work should report registration error, task-specific benefit, human-factors performance, and safety endpoints (51, 52).

Computer vision may eventually assist interpretation of dissection planes, but visible tissue segmentation does not establish microscopic tumor-margin clearance. Explanatory visualizations such as Grad-CAM may help explore model behavior (53); a heat map alone does not demonstrate anatomical correctness or causal reasoning (54). Surgical evaluation should prioritize task-specific accuracy, latency, failure recognition, and whether the display improves rather than distracts from operative judgment. Prospective testing should measure end-to-end display latency, false-alert burden and surgeon workload, and assess whether uncertain overlays should be suppressed or explicitly flagged. Acceptable performance should be established for the intended task rather than assumed from an arbitrary millisecond cutoff.

### 5.2 Prediction of postoperative complications

Postoperative complications after esophagectomy remain a major contributor to morbidity, mortality, and healthcare resource utilization. Pneumonia and anastomotic leakage are among the clinically consequential complications that motivate early-risk assessment, but their incidence varies by patient mix, procedure, definitions, and reporting system (6, 7). Earlier and more accurate prediction could support pre-emptive clinical intervention only if it is validated in the intended setting and linked to an effective action.

Van de Beld et al. compared temporal unimodal and multimodal models in 417 esophagectomy patients using repeated fourfold cross-validation (55). The best one-day-ahead leak AUC of 0.87 belonged to the temporal laboratory model, not multimodal fusion; the best pneumonia AUC was 0.74. These lead times are relative to recorded complication onset, not postoperative day 1. Jung et al. studied 864 patients using threefold cross-validation, with approximately two-thirds used for training in each fold, and reported neural-network AUCs of 0.672, 0.695 and 0.653 for major, medical and surgical complications (56). Their workflow figure places preprocessing before fold assignment, raising a potential leakage concern that requires implementation-level clarification. These internally evaluated models do not establish outcome benefit from model-guided management.

The intended benefit of postoperative prediction is earlier recognition linked to an effective intervention, not an alert alone. A model predicting an event one day before its recorded onset is not necessarily a model usable on postoperative day 1. Future studies should specify the prediction timestamp, available inputs, onset definition, and required lead time. Chest imaging used both as an input and in a pneumonia definition requires particular scrutiny for temporal or diagnostic-information leakage (55). Prespecified responses to alerts should be evaluated for missed events, false alarms, unnecessary investigations, clinician workload, and patient outcomes. Thresholds require local calibration and prospective evaluation within the intended pathway.

Preoperative risk prediction may support patient selection and informed consent, but useful individual risk estimates require calibration in the intended population. The modest internal discrimination reported for complication prediction (56) does not establish improved shared decision-making or a validated threshold for declining esophagectomy. Evaluation should compare an AI-supported discussion with existing clinical risk assessment and measure patient understanding, decisions, and subsequent outcomes.

### 5.3 AI at surgical decision points

Four surgical questions organize the conceptual framework: additional treatment after endoscopic resection, surgery versus active surveillance after cCR following nCRT, lymphadenectomy extent, and salvage-surgery selection. **Figure 2** groups evaluation into three complementary domains: model development and evaluation; transportability and potential utility; and prospective clinical impact. These domains are not evidence grades or sequential adoption criteria. The evaluation required depends on the proposed action, the consequences of false reassurance and unnecessary treatment, and opportunities for monitoring and rescue.

**Figure 2:**
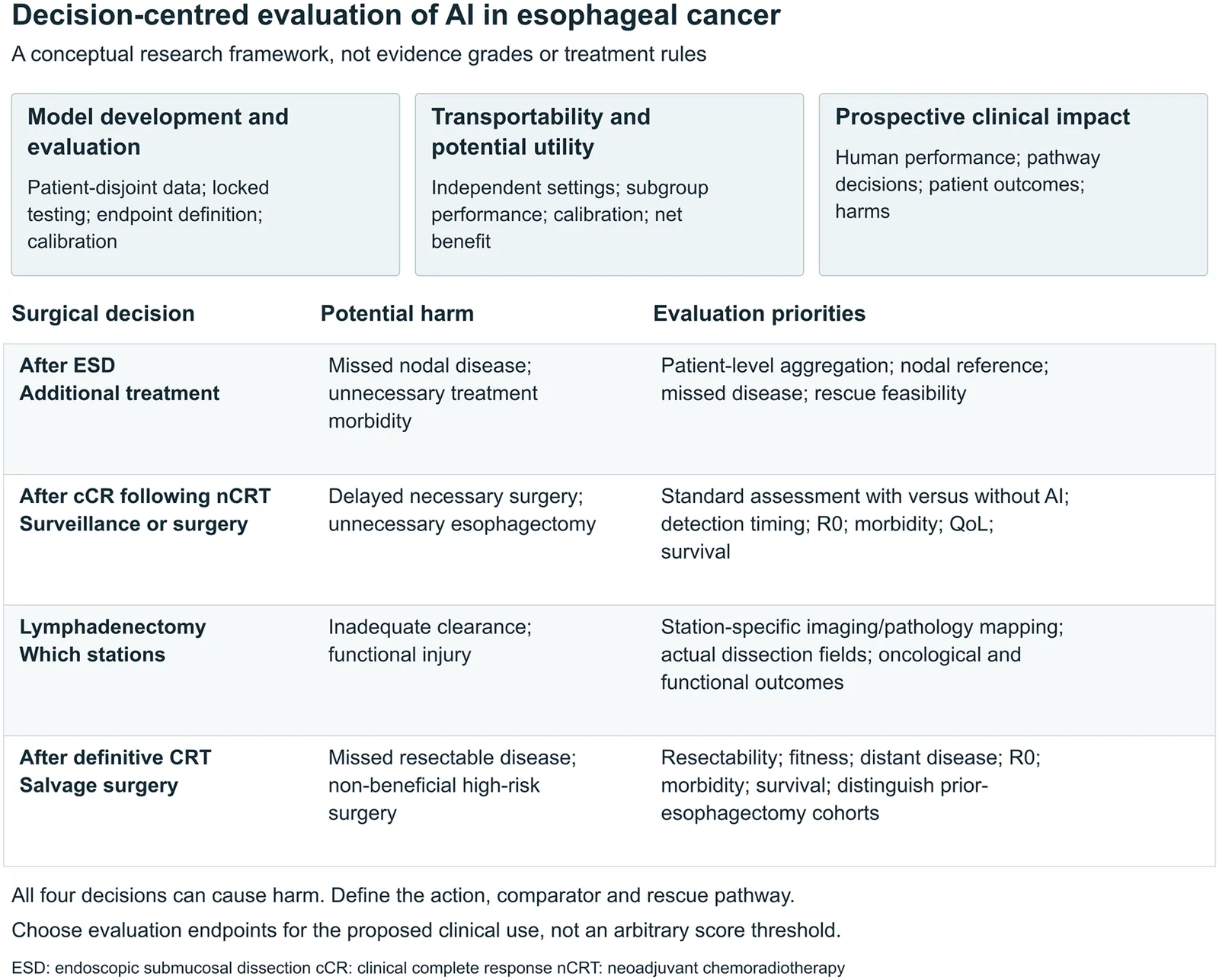
Author-proposed conceptual framework linking evaluation domains to four surgical decisions. The three domains are complementary, not evidence grades or clinical adoption criteria. Evaluation should address asymmetric harms: missed disease or delayed rescue, and unnecessary surgery with perioperative morbidity and long-term quality-of-life burden. The matrix gives research questions and outcomes, not treatment recommendations or numerical safety thresholds. The cCR scenario is restricted to assessment after neoadjuvant chemoradiotherapy (nCRT), not chemotherapy or chemoimmunotherapy; the salvage scenario concerns prior definitive chemoradiotherapy (CRT). Calibration applies throughout probability-model evaluation. ESD, endoscopic submucosal dissection; cCR, clinical complete response; R0, microscopically margin-negative resection; QoL, quality of life.

The first decision concerns additional treatment after endoscopic resection of early disease. Ren et al. illustrate the difference between slide-level model performance and the evidence needed for patient-level treatment allocation (22). The external cohort contained only four LNM-positive patients, with nodal status established using different reference methods. This is relevant risk-prediction evidence, but an AI-guided decision to add or omit treatment has not been tested. The next step is comparison with established pathological assessment using a locked patient-level decision policy. Relevant prospective outcomes include missed nodal disease, unnecessary additional treatment, morbidity, and the feasibility of timely rescue. No numerical NPV or risk cutoff for surgery or surveillance is recommended.

The second decision concerns active surveillance after cCR following nCRT. In SANO, protocolized active surveillance was non-inferior to standard surgery for two-year overall survival in selected patients with cCR (57). This result evaluates a complete surveillance-and-surgery-as-needed pathway, not a single test that perfectly excludes residual disease. SANO supplies direct evidence for the clinical pathway; response-prediction models provide adjacent evidence, not validation of AI-based eligibility. The missing comparison is the same surveillance pathway with versus without AI-supported selection. AI studies should compare added value over the existing response-assessment pathway and evaluate missed disease, time to detection, R0 resection when surgery is required, morbidity, quality of life, and longer-term survival. ctDNA and imaging/endoscopic features are complementary investigational inputs; neither an isolated NPV target nor ctDNA negativity establishes pathway safety (39–41).

The third decision concerns lymphadenectomy extent. The closest model evidence in this review is patient-level LNM or high nodal-burden prediction (22, 28). It does not establish which stations contain disease or whether changing clearance produces net benefit. Station-specific prediction linked to actual surgical decisions is the next evidentiary step. Models intended for this task should include nodal stations irrespective of PET positivity, with a prespecified mapping between imaging, dissection records, and station-specific pathology. The anatomical reference system, handling of unsampled stations, and treatment-related changes should be documented. Any proposal to omit or extend dissection requires evaluation of both oncological outcomes and functional harm rather than nodal-risk discrimination alone.

The fourth decision concerns salvage esophagectomy for persistent or recurrent locoregional disease after definitive CRT. Selection would require assessment of resectability, distant disease, patient fitness, expected morbidity, and potential benefit. This population should be distinguished from patients undergoing treatment for recurrence after a previous esophagectomy; prediction of response to salvage CRT after surgery (58) is not evidence for selecting salvage esophagectomy after definitive CRT. Among the studies examined here, no directly evaluated AI-guided salvage-esophagectomy selection pathway was identified. The immediate priority is a well-defined cohort with assessment of resectability and fitness, explicit treatment comparators, and morbidity and survival outcomes. Collaborative data collection should address these clinical definitions before a training architecture is chosen.

For each application, study protocols should define the patient population, prediction time, target condition, comparator, proposed action, and override or rescue pathway. Decision thresholds should be prespecified for prospective evaluation and justified by the intended benefit-harm trade-off; none is supplied here as a current clinical rule. If the positive class is residual disease, a false negative means missed residual disease; if the positive class is pCR, the dangerous error may instead be a false positive. Explicit label definitions are therefore essential. Clinical utility should be evaluated for the entire AI-supported pathway, including patient preferences and clinician decisions, rather than inferred from an isolated model score.

### 5.4 Esophageal cancer context for model application

Histology and treatment context affect model applicability. ESCC and EAC have different epidemiological and clinical contexts (2, 4). Reports should specify histology, tumor location and treatment pathway, and examine performance across these strata rather than assume that a change of institution alone establishes transportability.

For lymphadenectomy research, the intended action should be defined by the actual nodal stations dissected and the pathological reference available for those stations. Broad procedure labels alone are insufficient for evaluating a station-specific model. The proposed comparison should measure both cancer control and functional harm.

After nCRT, preSANO evaluated combined response assessment and SANO evaluated repeated assessment with surgery when indicated (39, 57). These studies make the timing and combination of tests central to interpretation. A model using pretreatment images and a model assessing post-treatment residual disease require different reference standards and clinical comparisons.

For image-derived models, segmentation and feature stability require attention (23, 59), alongside the regimen-specific response definitions discussed in Section 4.2. Performance on baseline imaging should not be transferred to post-treatment decisions without testing in that setting.

### 5.5 Surgical training and quality assessment

Phase recognition and structured video annotation provide candidate measures for training and feedback (47, 49, 60). The video-observer results described in Section 5.1 address recognition assistance, but do not establish durable skill acquisition or credentialing validity. Training studies should compare feedback strategies and assess retention and transfer to new cases before such measures are used for performance certification.

## 6 Prognostic modeling and post-recurrence treatment

Radiomics prognostic modeling after definitive chemoradiotherapy has been reported in proximal EC (61). Ye et al. combined radiomics and transcriptomics to explore radiotherapy sensitivity and prognosis, with experimental work on molecular targets including STUB1 (62). These studies address post-CRT risk or treatment sensitivity, not a validated postoperative surveillance schedule. Their relevance to follow-up depends on whether a risk estimate adds useful information beyond routine clinicopathological assessment at a defined time point.

Gu et al. studied response to salvage chemoradiotherapy for locoregional lymph-node recurrence after curative esophagectomy (58). This is prediction after recurrence has been recognized, not early recurrence detection or selection for salvage esophagectomy. ctDNA studies provide complementary clinical context (40, 41), but are not themselves evidence that an AI surveillance strategy improves outcomes.

## 7 Challenges and limitations

### 7.1 Data quality and standardization

Performance of any AI model is fundamentally constrained by the quality and representativeness of its training data. Many studies in the current EC AI literature are limited by small sample sizes, single-center data collection, selection bias, and inconsistent acquisition protocols (63, 64). Variation in imaging parameters, endoscopic equipment, and histopathological processing across institutions introduces sources of variability that affect model performance in ways that are not fully addressed by internal validation. International standardization initiatives, such as the Image Biomarker Standardisation Initiative (IBSI), provide important infrastructure for addressing these issues (59); consistent adoption across the field remains uneven.

### 7.2 External validation and the “AI chasm”

Independent external validation is necessary to assess transportability, but it does not by itself establish clinical benefit (65, 66). For probability-based decisions, reports should include calibration-in-the-large, calibration slope, calibration plots, and uncertainty intervals alongside discrimination (67). For illustration, a risk estimate that systematically overpredicts nodal disease could increase unnecessary treatment even if its ranking of patients is good. Decision-curve analysis can compare model-based net benefit with relevant strategies over clinically defensible threshold ranges (68). The threshold must correspond to a stated action and benefit-harm trade-off; a favorable decision curve is not a validated treatment cutoff or a substitute for prospective pathway evaluation. External assessment should also examine histology, regimen, center, and patient subgroups, followed by monitoring for calibration drift during use. Calibration should be assessed during internal as well as external evaluation when predicted probabilities are reported. Estimated net benefit is an analytic assessment of potential utility, not evidence that implementation improves patient outcomes.

### 7.3 Interpretability and the black-box problem

Interpretability may help clinicians examine model behavior, but explanations are not evidence that a prediction is correct (54, 69). Grad-CAM, SHAP, and LIME can support exploratory inspection, with limitations that depend on the model and explanation method (53, 70). Clinical evaluation should test whether the information presented enables appropriate reliance, recognition of errors, and safe override. External validity, calibration, and demonstrated task-specific benefit remain necessary even when a model produces a plausible explanation.

### 7.4 Methodological reporting and risk-of-bias assessment

Reporting and appraisal should match the study question: TRIPOD+AI addresses clinical prediction-model reporting, PROBAST+AI addresses quality, risk of bias and applicability, STARD-AI addresses diagnostic accuracy, and CLAIM addresses medical imaging AI (71–74). MI-CLAIM provides broader clinical modeling guidance (75). SPIRIT-AI, CONSORT-AI, and DECIDE-AI address protocols, trial reports, and early clinical evaluation, respectively (76–78). **Table 2** distinguishes their purposes. A completed reporting checklist cannot substitute for low risk of bias, external validation, or evidence of clinical utility.

**Table 2:** AI-specific reporting and risk-of-bias frameworks relevant to AI research in esophageal cancer.

Standard	Full Name/Focus	Year	Checklist structure	Primary Application
TRIPOD+AI (71)	Updated reporting guidance for clinical prediction models using regression or machine learning	2024	27 items, including subitems	Reporting of AI/ML prediction model studies (development and validation).
PROBAST+AI (72)	PROBAST+AI: quality, risk-of-bias, and applicability assessment for prediction models using regression or AI	2025	Separate development and evaluation assessments across four domains	Risk-of-bias assessment of AI/ML prediction model studies.
CLAIM (74)	Checklist for Artificial Intelligence in Medical Imaging	2024 update	44 items	Reporting of medical imaging–based AI studies, including radiomics.
MI-CLAIM (75)	Minimum Information about Clinical Artificial Intelligence Modelling	2020	6-part checklist	Reporting of broader clinical AI studies, including non-imaging data.
STARD-AI (73)	Standards for Reporting Diagnostic Accuracy Studies - AI	2025	40 items	Diagnostic-accuracy studies of AI-based tests.
SPIRIT-AI (76)	SPIRIT extension for clinical trial protocols involving AI	2020	15 extension items	Trial protocols for AI interventions.
CONSORT-AI (77)	CONSORT extension for clinical trial reports involving AI	2020	14 extension items	Completed RCTs of AI interventions.
DECIDE-AI (78)	Developmental and Exploratory Clinical Investigation of DEcision support systems driven by AI	2022	27 items	Early-stage clinical evaluation of AI-based decision-support systems.

AI, artificial intelligence; ML, machine learning; RCT, randomized controlled trial. Versions and structures refer to the cited publications. Reporting completeness, risk of bias, and clinical readiness are separate assessments.

### 7.5 Ethical, legal, and regulatory considerations

Integration of AI into clinical practice raises ongoing questions of accountability, data privacy, algorithmic bias, and informed consent (79), and regulatory frameworks for medical AI continue to evolve (80). These are well-discussed in general AI ethics literature and we do not extend that discussion here; the EC-specific point worth retaining is that responsibility for decisions taken with AI input should remain clearly assigned to the responsible clinician, particularly at the high-consequence surgical inflection points discussed in Section 5.3.

### 7.6 Infrastructure and implementation barriers

Implementation requires secure data handling, interoperability, reliable computing, and a defined clinical workflow (65, 81). Training should address intended use, uncertainty, failure modes, and override conditions. Resource requirements depend on the application; an institution should evaluate integration costs, maintenance, and effects on access rather than assume that technical performance alone will produce sustainable benefit.

### 7.7 Challenges of multimodal data integration in EC research

Multimodal AI research in EC depends on accurate patient linkage, consistent predictor definitions, and separation of model development from evaluation. The following points are methodological considerations, not estimates of how frequently these problems occur in the literature reviewed.

First, patient-level identifier reconciliation across data sources is required for any multimodal EC dataset, which typically integrates endoscopy, radiology, pathology, operative, treatment, and follow-up records. Each source system may use distinct identifiers, and linkage errors can contaminate features, labels, or development-test separation. The frequency of such errors is setting-dependent and should not be generalized from isolated studies. EC AI reports should instead disclose the linkage method, audit process, residual uncertainty, and whether linkage was performed before patient-level data splitting (82, 83).

Second, clinical variables recorded as free text need consistent definitions before use as model predictors. Neoadjuvant treatment records may describe drug regimens, radiation dose and fractionation, and treatment timing differently across centers. Extraction can use natural-language processing, manual review, or a combination, but its accuracy and the handling of ambiguous or conflicting records should be documented. A model may otherwise exploit documentation patterns associated with a source system rather than the intended clinical variables. This is a potential source of limited transportability, not a demonstrated explanation for every failure of external validation.

Third, radiomics pipelines should report the candidate feature space, segmentation and feature stability, missing-data handling, and model complexity relative to patient and event counts (23, 59, 71, 72). Data-dependent preprocessing, including imputation and harmonization, should be fitted only on the relevant training partition and then applied to its evaluation partition. Feature selection and hyperparameter tuning should likewise remain within development data; nested resampling is appropriate when model selection and internal performance estimation use resampling. A final test set should remain untouched until the pipeline is locked. Neither a high AUC nor a small final feature set alone demonstrates overfitting. Assessment instead requires examination of the full development process, uncertainty in performance estimates, calibration where probabilities are predicted, and evaluation on independent patients. Poor external performance may also reflect differences in case mix or measurement rather than overfitting alone. No universal AUC or feature-count threshold is proposed here.

Leakage can arise when predictors are generated after the intended prediction time or contain information used to define the outcome. The postoperative imaging example and the registry metastatic-status model illustrate why predictor availability and label construction must be examined directly (29, 55). Poor external performance alone is not proof of leakage. AI-generated clinical text raises a separate, emerging provenance question; an adverse effect on EC model performance has not been established, so provenance logging should be presented as a reporting proposal rather than evidence of clinical model collapse.

A concise data-provenance disclosure could therefore record the patient-linkage procedure and audit, extraction and validation of free-text variables, the order of splitting and preprocessing, predictor timestamps relative to the outcome, and any known AI-generated source content. These are proposed reporting practices, not empirically validated requirements unique to EC. They complement the established reporting and appraisal frameworks in **Table 2**.

## 8 Priorities for clinical evaluation

### 8.1 Prospective nodal mapping and decision studies

A prospective multicenter cohort could link preoperative imaging to a common map of dissected nodal stations and pathology. A locked model should first be evaluated without changing the operation, before a controlled study tests a specified change in dissection. Multimodal or foundation-model components should earn inclusion through incremental value, not the number of data sources (84, 85). Federated learning is an optional way to support collaboration, not a substitute for shared clinical definitions (86). Source-linked language-model extraction could be evaluated for assembling these records, with errors checked against the clinical source before use (87, 88).

### 8.2 Controlled evaluation of intraoperative assistance

Recognition assistance should progress from held-out video-observer evaluation to controlled use during surgery. Informed by the Furube study (50), early evaluation can measure correct recognition, response time and inappropriate reliance; operative evaluation should additionally measure false alerts, latency, workflow disruption and complications. Training and test videos must remain patient-disjoint, and analysis should account for repeated observations from the same surgeon or case. This separates a useful display from a claim of reduced surgical injury (51, 52, 78).

### 8.3 Regimen-specific response assessment studies

For patients assessed after nCRT, a prospective study could compare standard multimodal response evaluation with the same assessment plus a locked AI component. It should distinguish primary and nodal residual disease and report missed disease, subsequent detection and rescue-treatment outcomes. Chemotherapy and chemoimmunotherapy require separate cohorts and response definitions; ctDNA should be evaluated for incremental value rather than treated as a stand-alone exclusion test (38–41). Protocol and trial reporting should follow the appropriate AI extensions (76–78). Medical-imaging methods and CT-based adaptive-radiotherapy work in head and neck cancer offer implementation lessons (25, 89), but do not supply EC surgical efficacy evidence.

## 9 Conclusions

The selected studies illustrate why clinical task and evaluation unit matter. A randomized trial demonstrated improved lesion detection in its study setting; nodal and response models provide predictive evidence, and video studies are beginning to examine human performance. Within this selective evidence base, clinical benefit from AI-guided esophagectomy, organ-preservation, lymphadenectomy or salvage decisions has not been established. This conclusion is not an exhaustive assessment of every EC AI study.

The next priority is to test a defined contribution to an existing care pathway. Clinically useful evaluation must connect model output to a specified action, comparator and patient outcome, while preserving clinician override and timely rescue.
