# Supplementary material for: Artificial intelligence in esophageal cancer: a narrative review from a thoracic surgical perspective
Source: Front Oncol. 2026 Oct 20;16:1917622. doi: 10.3389/fonc.2026.1917622 (PMC13728921; doi:10.3389/fonc.2026.1917622)
Supplement: Supplementary file 1 [file DataSheet1.pdf]

Supplementary Methods

Documented revision searches

This supplement documents the PubMed and Web of Science searches conducted during revision on 6 September 2026. These searches supplement the narrative review; the original search history was unavailable. Embase was not accessed.

PubMed strategies were retained in execution records. Web of Science strategies are the author-confirmed query text, supported by result screenshots and bibliographic exports. W2 used W1 with the publication-date range replaced. The selected Web of Science indexes and any additional interface filters were not recorded.

PubMed execution

PubMed was queried using NCBI E-utilities. S1 covered publication dates from 1 January 2015 to 6 September 2026; S2 used 1 April to 6 September 2026. S3 used the latter interval for clinical context without requiring AI terms. All three submitted queries restricted language to English. The saved query records contain no human-only, study-design or full-text-availability filter. Exact submitted queries, server translations, timestamps, ESearch responses, PMID lists and EFetch XML were retained in the original archive. UTC start times were 14:38:33 for S1, 14:38:46 for S2 and 14:38:59 for S3 (22:38 on 6 September in China Standard Time).

Table S1 Retrieval accounting

Database or operation	Set	Records
PubMed full-period AI	S1	1786
PubMed AI update	S2	172
PubMed clinical-context update	S3	162
PubMed S2/S3 overlap	Intersection	10
PubMed update union	S2 union S3	324
PubMed full union	S1 union S3	1938
Web of Science full-period AI	W1	1976
Web of Science AI update	W2	156
Web of Science clinical-context update	W3	138
Web of Science W2/W3 overlap	Intersection	9
Web of Science update union	W2 union W3	285
Web of Science full union	W1 union W3	2105
Combined database-level unique records	Before cross-database matching	4043
Collapsed records in preliminary matching	Not study exclusions	1480
Preliminary combined record clusters	Full pool	2563
Clusters with at least one update-set record	Update working pool	387

S2 is a subset of S1, and W2 is a subset of W1; adding each subset to its parent would double-count records. W1 was exported in batches of 1000 and 977 records with one shared accession number, yielding 1976 unique records.

TXT and RIS accession sets agreed. Cross-database counts are provisional record-cluster counts, not independent studies or patient cohorts.

Record handling and screening boundaries

Records were matched by PMID, DOI, and then exact normalized title with year and first author. Matching collapsed 1268 record links by PMID, 205 by DOI and 7 by title/year/author. Fifteen ambiguous matches were retained separately. Near-title matches and multiple reports from one patient cohort were not exhaustively resolved; Table S1 therefore reports preliminary record clusters rather than included-study counts.

AI-assisted preliminary triage covered the 387 update clusters using titles and bibliographic information, with abstract reading recorded for 132. Fifty clusters lacked an abstract in both exports. Initial categories were 83 AI original-study candidates, 113 clinical-context candidates, 88 review/method/context records, 12 unresolved records, 32 non-complete-result reports, 4 corrections and 55 topic/scope exclusions. These were preliminary categories rather than final inclusions. Selected candidates subsequently underwent targeted source checks. The remaining 2176 clusters in the broad pool were not fully screened. Independent duplicate human screening and a comprehensive formal risk-of-bias assessment were not performed.

The review considers ESCC, EAC and relevant precursor lesions across detection, staging, treatment response, perioperative care and related treatment workflows. Clinical treatment, ctDNA and surveillance studies provide context rather than AI performance evidence. Reviews, protocols, meeting reports and corrections are not counted as independent completed clinical AI studies. Full-text inaccessibility was not an automatic topic exclusion. Table 1 selection was purposive, based on clinical relevance, task coverage and source verifiability, not ranked AUC. Abstract-only sources and unconfirmed methodological details are identified. For Li 2024, the full report was obtained and its patient-level primary detection endpoint checked against Table 2 of Li et al. [14] and the Methods; supplementary materials were not available.

Publication-date fields and issue dates do not necessarily identify first online publication. Update records are therefore not automatically newly published studies, nor were they compared with a verified historical screened library. The original raw exports remain unchanged. Identified extraction or abstract-assignment discrepancies are recorded in working audit files, rather than silently replacing the source archive.

Exact submitted PubMed queries

S1 full AI

```
("Esophageal Neoplasms"[MeSH Terms] OR ((esophag*[Title/Abstract] OR oesophag*[Title/Abstract]) AND
(cancer*[Title/Abstract] OR neoplas*[Title/Abstract] OR carcinoma*[Title/Abstract])) OR
ESCC[Title/Abstract] OR "Barrett Esophagus"[MeSH Terms] OR Barrett*[Title/Abstract] OR
esophagectom*[Title/Abstract] OR oesophagectom*[Title/Abstract]) AND ("Artificial Intelligence"[MeSH
Terms] OR "Machine Learning"[MeSH Terms] OR "Deep Learning"[MeSH Terms] OR "artificial
intelligence"[Title/Abstract] OR "machine learning"[Title/Abstract] OR "deep learning"[Title/Abstract]
OR "neural network*"[Title/Abstract] OR radiomic*[Title/Abstract] OR "computer aided"[Title/Abstract]
OR "computer assisted diagnosis"[Title/Abstract] OR "computer vision"[Title/Abstract] OR "natural
language processing"[Title/Abstract] OR "large language model*"[Title/Abstract] OR "foundation
model*"[Title/Abstract]) AND ("2015/01/01"[Date - Publication] : "2026/09/06"[Date - Publication])
AND english[Language]
```

S2 update AI

```
("Esophageal Neoplasms"[MeSH Terms] OR ((esophag*[Title/Abstract] OR oesophag*[Title/Abstract]) AND
(cancer*[Title/Abstract] OR neoplas*[Title/Abstract] OR carcinoma*[Title/Abstract])) OR
ESCC[Title/Abstract] OR "Barrett Esophagus"[MeSH Terms] OR Barrett*[Title/Abstract] OR
```

```
esophagectom*[Title/Abstract] OR oesophagectom*[Title/Abstract]) AND ("Artificial Intelligence"[MeSH
Terms] OR "Machine Learning"[MeSH Terms] OR "Deep Learning"[MeSH Terms] OR "artificial
intelligence"[Title/Abstract] OR "machine learning"[Title/Abstract] OR "deep learning"[Title/Abstract]
OR "neural network*"[Title/Abstract] OR radiomic*[Title/Abstract] OR "computer aided"[Title/Abstract]
OR "computer assisted diagnosis"[Title/Abstract] OR "computer vision"[Title/Abstract] OR "natural
language processing"[Title/Abstract] OR "large language model*"[Title/Abstract] OR "foundation
model*"[Title/Abstract]) AND ("2026/04/01"[Date - Publication] : "2026/09/06"[Date - Publication])
AND english[Language]
```

S3 update clinical

```
("Esophageal Neoplasms"[MeSH Terms] OR ((esophag*[Title/Abstract] OR oesophag*[Title/Abstract]) AND
(cancer*[Title/Abstract] OR neoplas*[Title/Abstract] OR carcinoma*[Title/Abstract])) OR
ESCC[Title/Abstract] OR "Barrett Esophagus"[MeSH Terms] OR Barrett*[Title/Abstract] OR
esophagectom*[Title/Abstract] OR oesophagectom*[Title/Abstract]) AND ((neoadjuvant[Title/Abstract]
AND (immunotherap*[Title/Abstract] OR chemoimmunotherap*[Title/Abstract] OR "immune
checkpoint"[Title/Abstract])) OR ctDNA[Title/Abstract] OR "circulating tumor DNA"[Title/Abstract] OR
"circulating tumour DNA"[Title/Abstract] OR "active surveillance"[Title/Abstract] OR "clinical
complete response"[Title/Abstract]) AND ("2026/04/01"[Date - Publication] : "2026/09/06"[Date -
Publication]) AND english[Language]
```

Web of Science strategies confirmed by the author

The supplied instructions specified Web of Science Core Collection, advanced Topic search (TS), English language (LA), and publication-date ranges (DOP). Index selection and additional interface settings are not inferred from these instructions.

W1

```
TS=(
  (
    ((esophag* OR oesophag*) AND (cancer* OR neoplas* OR carcinoma*))
    OR ESCC
    OR Barrett*
    OR esophagectom*
    OR oesophagectom*
  )
  AND
  (
    "artificial intelligence"
    OR "machine learning"
    OR "deep learning"
    OR "neural network*"
    OR radiomic*
    OR "computer aided"
    OR "computer assisted diagnosis"
    OR "computer vision"
    OR "natural language processing"
    OR "large language model*"
    OR "foundation model*"
  )
)
AND LA=(English)
AND DOP=2015-01-01/2026-09-06
```

W2

```
TS=(
  (
    ((esophag* OR oesophag*) AND (cancer* OR neoplas* OR carcinoma*))
    OR ESCC
    OR Barrett*
    OR esophagectom*
    OR oesophagectom*
  )
  AND
  (
    "artificial intelligence"
    OR "machine learning"
    OR "deep learning"
    OR "neural network*"
    OR radiomic*
    OR "computer aided"
    OR "computer assisted diagnosis"
    OR "computer vision"
    OR "natural language processing"
    OR "large language model*"
    OR "foundation model*"
  )
)
AND LA=(English)
AND DOP=2026-04-01/2026-09-06
```

W3

```
TS=(
  (
    ((esophag* OR oesophag*) AND (cancer* OR neoplas* OR carcinoma*))
    OR ESCC
    OR Barrett*
    OR esophagectom*
    OR oesophagectom*
  )
  AND
  (
    (
      neoadjuvant
      AND
      (
        immunotherap*
        OR chemoimmunotherap*
        OR "immune checkpoint"
      )
    )
    OR ctDNA
    OR "circulating tumor DNA"
    OR "circulating tumour DNA"
    OR "active surveillance"
    OR "clinical complete response"
  )
)
AND LA=(English)
AND DOP=2026-04-01/2026-09-06
```

Documentation

PubMed date-field interpretation: https://pubmed.ncbi.nlm.nih.gov/help/

Web of Science search and record-export documentation: https://webofscience.help.clarivate.com/en-us/Content/document-search.htm and https://webofscience.help.clarivate.com/en-us/Content/export-records.htm

Study selection and source checks

Table S2 links the 14 representative studies to their role, source-check scope and manuscript location. It is a purposive selection map, not a complete disposition list for all 2563 record clusters or 387 update clusters. Additional candidate triage notes are retained as internal working records and are not presented as confirmed study defects. Source access and author review are separate: review of available material does not establish verification of unavailable appendices or code. The examples were chosen to illuminate the clinical questions and validation issues emphasised in this review. Their distribution does not estimate the distribution or maturity of research across the field.

Dongrong Peng reviewed the 14 representative studies, key clinical evidence and identified reporting discrepancies on 9 September 2026, using available source materials. This did not constitute exhaustive or independent duplicate screening. Preliminary classifications of the wider search pool were not all systematically reassessed. Abstract reading was recorded for 132 update clusters; other classifications remained preliminary bibliographic triage.

Table S2 Study selection map

Representative studies retained in Table 1

Study and task	Selection decision and rationale	Source check scope	Location	Author review
Li 2024 [14]; Screening	Retained for its randomised patient-level detection endpoint. Main report checked; subgroup estimates not used because of internal reporting inconsistencies.	Full report: development overview (p. 2); Table 2 of Li et al. [14] (p. 5); verification limitations (p. 8); trial methods (p. 9). Three-centre system development used 191,744 esophagogastroscopic images; detailed partitions and patient counts remain unverified.	3.1; Table 1	Dongrong Peng 9 September 2026
Yuan 2022 [15]; Endoscopic diagnosis	Multi-modality detection with external image testing	Scanned article pp. 2, 4–5; Tables 1–2	3.1; Table 1	Dongrong Peng 9 September 2026
Ren 2025 [22]; Histopathology	T1 nodal risk linked to post-ESD treatment; slide/case distinction	Article pp. 2–5; Tables 3–4	3.2; 5.3; Table 1	Dongrong Peng 9 September 2026
Bouzid 2024 [20]; Histopathology	Non-endoscopic Barrett screening and separate cohort testing	Article Table 1, results and Table 3	3.1; Table 1	Dongrong Peng 9 September 2026
Shen 2018 [26]; Staging	Temporal validation; signature distinguished from nomogram	Article pp. 1, 5–7; Figure 4	4.1; Table 1	Dongrong Peng 9 September 2026
Chen 2022 [27]; Staging	Model-specific nodal evaluation; selection among compared models	Article Methods and Table 3	4.1; Table 1	Dongrong Peng 9 September 2026
Li 2026 [28]; Nodal staging	N2+ burden with temporal/geographic testing; C0332	Accessible original report; Table 1 extraction	4.1; Table 1	Dongrong Peng 9 September 2026
Kasai 2024 [42]; Treatment response	Same-hospital departmental split; endpoint distinguished from pCR	Article Methods and results pp. 6–7	4.2; Table 1	Dongrong Peng 9 September 2026
Xie 2023 [43]; Treatment response	Radiographic response; image/patient aggregation unresolved	Article Figure 1 and results pp. 5–6	4.2; Table 1	Dongrong Peng 9 September 2026

Study and task	Selection decision and rationale	Source check scope	Location	Author review
Hu 2020 [35]; Treatment response	Resection-specimen pCR and external testing	Supplement p. 14; repository Methods and abstract	4.2; Table 1	Dongrong Peng 9 September 2026
van Jaarsveld 2026 [48]; Intraoperative surgery	Video and patient partitions separately reported; C0068	Accessible original report; separate anatomy/phase extraction	5.1; Table 1	Dongrong Peng 9 September 2026
van de Beld 2024 [55]; Postoperative complications	Time-dependent complication prediction; correct best model attribution	Article abstract and results pp. 7–11	5.2; Table 1	Dongrong Peng 9 September 2026
Jung 2023 [56]; Postoperative complications	Complication prediction; CV/preprocessing interpretation	Article Methods and Figures 1–2	5.2; Table 1	Dongrong Peng 9 September 2026
Wan 2024 [29]; Metastasis prediction	Baseline metastatic status; illustrates prediction-time limitations	Article pp. 1, 3–5	4.1; Table 1	Dongrong Peng 9 September 2026

Li 2024 source interpretation

The intention-to-treat result retained in Table 1 is concordant between the abstract, Results and Table 2 of Li et al. [14]: 28/1556 versus 14/1561, P=0.029. The report defines the primary endpoint as the proportion of patients with at least one high-risk lesion, including low- or high-grade intraepithelial neoplasia and cancer. Table 2 of Li et al. [14] reports per-protocol P=0.021, whereas the Discussion reports P=0.017; Table 3 also contains subgroup denominators that do not reconcile with the randomised totals. These secondary/subgroup findings were not used to strengthen the review conclusions. The article acknowledges that non-biopsied examinations could be classified as true negatives, so diagnostic accuracy is not treated as an unbiased estimate of missed disease. Supplementary model-development methods and the separate study protocol were not available for verification.

Generative AI assistance

ChatGPT and Codex (OpenAI; https://openai.com/) assisted preliminary bibliographic triage and data extraction, as well as drafting, editing and conceptual figure preparation. Exact model identifiers and software versions were not retained. Author verification and screening boundaries are described above.

Source scope of narrative examples

The narrative accounts of Takeuchi 2022 (doi:10.1245/s10434-022-11996-1), Matsuda 2023 (doi:10.1245/s10434-023-13140-z) and Furube 2025 (doi:10.1245/s10434-025-17649-3) are based on published abstracts. Full methodological appraisal was therefore limited. Unclear patient-level partitioning is not treated as evidence that separation was absent, and no unreported randomisation of video presentation or reduction in surgical injury is inferred.
